# Inflammatory markers as independent predictors for stroke outcomes

**DOI:** 10.1002/brb3.1922

**Published:** 2020-12-12

**Authors:** Xiao‐Guang Zhang, Jie Xue, Wen‐Hao Yang, Xu‐Shen Xu, Hong‐Xian Sun, Liang Hu, Ling‐Yun Liu, Yun‐Hua Yue

**Affiliations:** ^1^ Department of Neurology Yangpu Hospital Tongji University School of Medicine Shanghai China

**Keywords:** inflammation, national institute of health stroke scale, small‐artery occlusion, stroke

## Abstract

**Background:**

Inflammation plays an important role in the pathophysiology of stroke. The aim of the present study was to investigate the association between various inflammatory risk markers and ischemic stroke outcome and subtype.

**Methods:**

A total of 3,013 ischemic stroke patients who were admitted to our hospital from 01/01/2016 to 12/30/2018 were retrospectively studied. Stroke subtypes were defined by the Trial of Org 10172 in Acute Stroke Treatment (TOAST) classification. Levels of five common inflammatory markers including white blood cell (WBC) count, neutrophil, lymphocyte, serum C‐reactive protein (CRP), and interleukin‐6 (IL‐6) were measured, and eleven conventional risk factors were further evaluated in the prediction of overall mortality as well as three functional outcomes defined by the National Institute of Health Stroke Scale (NIHSS), the modified Rankin Scale (mRS), and the Barthel Index (BI). Independent predictors of outcome were identified by multivariate logistic regression, and an importance score measured by the area under the receiver operating characteristics curve for each predictor using a Naive Bayes model was reported.

**Results:**

Neutrophil and WBC were significantly higher in large‐artery atherosclerosis (LAA) and cardioembolism (CE) subtype. In contrast, lymphocyte was significantly higher in small‐artery occlusion (SAO). Neutrophil–lymphocyte ratio and CRP level were the best independent predictors, after adjustment for traditional risk factors and TOAST subtype for all four types of outcomes.

**Conclusion:**

Inflammatory risk markers including neutrophil, lymphocyte, and CRP may have strong independent prediction values for stroke outcome.

## Graphical abstract

The aim of the present study was to investigate the association between various inflammatory risk markers and ischemic stroke outcome and subtype. We found that inflammatory risk markers including neutrophil, lymphocyte, and CRP may have strong independent prediction values for stroke outcome.

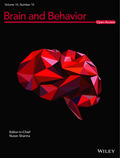

## 1 INTRODUCTION

Stroke is one of the leading causes of disability and death worldwide (Hankey, 2017). In China, the annual stroke mortality rate is approximately 157 per 100,000 people (Liu et al., 2011). Hypertension, dyslipidemia, and atrial fibrillation are known to be causal risk factors for stroke, and treatment of these conditions reduces the incidence of stroke. Cigarette smoking, alcohol abuse, and diabetes mellitus are also likely causal risk factors (Boehme et al., 2017). However, these conventional stroke risk factors do not fully account for the incidence of stroke, especially in young stroke victims (Lindsberg & Grau, 2003).

Chronic inflammation has been recently proposed to be an important risk factor for stroke (Lindsberg & Grau, 2003). A systemic inflammatory response occurs after ischemic stroke, either as part of the process of brain damage or in response to complications such as deep venous thrombosis. Although the presence of this inflammation is readily diagnosable via medical examinations such as computed tomography (CT), there is a notable gap between these objective measures and patient symptomatology, which makes the treatment and management of stroke challenging. Several studies have reported that higher levels of inflammatory markers such as CRP and IL‐6 are associated with worse outcome after ischemic stroke (Kuwashiro et al., 2013; Kwan et al., 2013; Park et al., 2013; Rajeshwar et al., 2012). Identifying predictors of functional outcome may be of assistance to physicians when confronted with these concerns from stroke patients. Improvement in the estimation of clinical outcomes could result in more specific management of stroke rehabilitation as well as clearer informing of patients and their relatives (Boehme et al., 2017). To this purpose, three main scoring systems including NIHSS, mRS, and BI are widely used for estimating the severity of stroke, functional impairment, and disability at onset and for assessing prognostic information in hospital (Ghandehari, 2013). These scaling systems have also been proposed in multiple studies to represent functional outcome; however, it is unclear which scaling systems scale is preferable (Harrison et al., 2013). Moreover, a systematic evaluation of common inflammatory risk factors in the prediction for mortality or functional outcomes defined by different scaling system is largely missing. Previous studies were often too small and did not adequately adjust for conventional risk confounders or etiological subtypes. We therefore aimed to investigate the association of five markers of acute inflammatory—CRP, IL‐6, WBC, neutrophil, and lymphocyte with four poor outcomes defined by overall mortality, NIHSS, mRS, and BI after ischemic stroke in a large retrospective cohort study of stroke patients. The addition of markers of inflammation to validated clinical prognostic models might improve the prediction of poor outcome after stroke.

## 2 SUBJECTS AND METHODS

### 2.1 Study population and outcome measures

This retrospective study comprises 3,013 acute ischemic stroke patients who were admitted to our hospital from 01/01/2016 to 12/30/2018. Etiological stroke subtypes were classified according to the Trial of Org 10172 in Acute Stroke Treatment (TOAST) system (Adams et al., 1993), including large‐artery atherosclerosis (LAA), small‐artery occlusion (SAO), and cardioembolism (CE). In addition to the primary end point measured by all‐cause mortality at 90 days, functional outcomes were assessed when patients arrived at the hospital with three popular measures: NIHSS, mRS, and BI (Kasner, 2006). Stroke severity was defined by using NIHSS system. Patients with a NIHSS score ≥21 were defined as having a poor outcome otherwise a good outcome (Harrison et al., 2013). mRS was used to evaluate the disability, with six grades from 0 to 5. Patients with a mRS score ≤2 were defined as having a good outcome, and patients with a mRS score >2 were defined as having a poor outcome (Harrison et al., 2013). The BI is a standard and well‐validated scale that measures independence in personal 10 basic activities of daily living, scoring 0–100 with 5‐point increments. BI ≥ 95 was defined as good outcome (Harrison et al., 2013). The study was approved by the ethics committee of Yangpu Hospital, Tongji University School of Medicine. A total of 11 conventional risk factors including age, gender, body mass index (BMI), smoking history, drinking history, history of diabetes, hypertension, atrial fibrillation (AF), coronary artery disease (CAD), systolic blood pressure (BP), and lipid level such as cholesterol, high‐density lipoprotein (HDL), and low‐density lipoprotein (LDL) were collected. The formula for BMI is weight in kilograms divided by height in meters squared. All participants gave written informed consent.

### 2.2 Laboratory measurements

Among stroke cases, blood sampling was performed within 24–48 hr of the stroke event. Venous blood samples were drawn between 8:30 and 10:30 a.m. after an overnight fast. CRP, total cholesterol, HDL, and LDL were analyzed with standard assays on aDxC 700 AU analyzer (Beckman Coulter). Counts of white blood cell (WBC), neutrophil, and lymphocyte were analyzed on an Advia 2120 analyzer (Siemens Diagnostics). IL‐6 was determined with chemiluminescent microparticle immunoassay on a MLX Microtiter luminometer (Dynex Technologies).

### 2.3 Statistical analysis

Demographic data were analyzed using descriptive methods, with the mean ± standard deviation (*SD*) or the median. Comparison of differences in the TOAST subtypes was made using *t* tests and analysis of variance (ANOVA). Spearman's rho was used to examine the correlation between numeric variables. For outcome association analysis using Bayesian and logistic models, we added patients with death into poor outcome defined by NIHSS, mRS, and BI. Missing values for laboratory measurements such as level of CRP, IL‐6, total cholesterol, HDL, LDL, counts of WBC, neutrophil, and lymphocyte were replaced by the median value in order to have the possibility of using all available clinical information in the regression model (Khosla et al., 2010). TOAST subtypes were further divided into nonlacunar (CE or LAA) and lacunar (SAO). A Naive Bayes classifier was used to compare the prediction power of risk factors by calculating the area under receiver operating characteristics curve (AUC) (Kim et al., 2019). Stepwise feature selection and multivariable logistic regression models were used to analyze all possible confounding factors for four types of outcomes. Variables associated with outcome in the univariate analysis with a *p*‐value of <.1 were included in a multivariate model. Odds ratios (ORs) and 95% confidence intervals (95% CIs) were calculated for these parameters. Logarithmically transformed CRP and IL‐6 levels were used in logistic regression model. Data were analyzed in R 3.5.0 environment. Statistical analyses were performed in a 2‐tailed fashion. A *p*‐value <.05 was considered to be statistically significant.

## 3 RESULTS

### 3.1 Characteristics of patients and TOAST subtype

Table 1 describes the baseline clinical characteristics of the overall population. This retrospective study included a total of 3,013 subjects with a mean age of 73 years (±13 years). Of them, 1801 (59.8%) were men and 1,212 (40.2%) were women. Among all patients, 998 (35.6%) were smokers, 377 (12.5%) had drinking history, 2,175 (77.6%) had a history of hypertension, 1,065 (38%) had a history of diabetes mellitus, 288 (10.27%) had a history of AF and 335 (11.95%) had history of CAD. The mean score of all the subjects for NIHSS was 4.65 (±6.07), for mRS was 2.11(±3.03), and for BI was 79.4(±24.28), respectively.

**Table 1:** Baseline characteristics of the overall population

Characteristic	Total, *n* = 3,013
Gender, male, number (%)	1,801 (59.8%)
Age, mean (*SD*)	72.9 (12.8)
BMI, mean (*SD*)	25.5 (17.6)
Systolic BP (Hg), mean (*SD*)	147 (20.5)
CRP (mg/L), mean (*SD*)	18.5 (37.1)
WBC (10^9^/L), mean (*SD*)	7.42 (2.72)
Neutrophil (%), mean (*SD*)	68.0 (12.3)
lymphocyte (%), mean (*SD*)	25.0 (10.2)
IL−6 (pg/ml), mean (*SD*)	52.8 (228)
Cholesterol (mM), mean (*SD*)	4.57 (1.19)
HDL(mM), mean (*SD*)	1.06 (0.290)
LDL(mM), mean (*SD*)	2.95 (0.888)
Smoking history, number (%)	
No	1,805 (59.9%)
Yes	998 (33.1%)
Drinking history, number (%)	
No	2,426 (80.5%)
Yes	377 (12.5%)
Hypertension history, number (%)	
No	628 (20.8%)
Yes	2,175 (72.2%)
Diabetes history, number (%)	
No	1,738 (57.7%)
Yes	1,065 (35.3%)
Atrial fibrillation history, number (%)	
No	2,515 (83.5%)
Yes	288 (9.6%)
Coronary artery disease history, number (%)	
No	2,468 (81.9%)
Yes	335 (11.1%)
NIHSS^a^, mean (*SD*)	4.65 (6.07)
BI^a^, mean (*SD*)	79.4 (24.3)
mRS^a^, mean (*SD*)	2.11 (3.03)
TOAST subtype, number (%)	
CE	175 (5.8%)
LAA	1,364 (45.3%)
SAO	1,077 (35.7%)

Abbreviations: BI, Barthel Index; BMI, body mass index; BP, blood pressure; CE, cardioembolism; CRP, C‐reactive protein; HDL, high‐density lipoprotein; IL, interlink; LDL, low‐density lipoprotein; mRS, modified Rankin Scale; NIHSS, National Institutes of Health Stroke Scale; SAO, small‐artery occlusion; *SD*, standard deviation; TOAST, the Trial of Org 10172 in Acute Stroke Treatment, LAA, large‐artery atherosclerosis; WBC, white blood cell.

^a^ Baseline score was measured when patients arrived at the hospital.

Table 2 describes the baseline clinical characteristics of the overall population classified by using TOAST etiologic classification method. Among 2,616 patients who had TOAST subtype information, 175 cases (6.69%) were CE subtype, 1,364 cases (52.14%) were LAA subtype, and 1,077 cases (41.17%) had SAO subtype. Counts of WBC and neutrophil, level of IL‐6, and CRP were significantly higher in CE or LAA, supporting the role of chronic inflammatory mechanism in stroke. Proportion of cases having history of hypertension or diabetes mellitus was significantly lower in patients with CE than in patients with SAO or LAA; in contrast, CE patients had highest frequency of AF history and CAD history, indicating AF and CAD may strongly contribute to the overall mortality of CE subtype. Other conventional risk factors such as age, smoking status, blood cholesterol, and LDL level also reached statistical significance among the three groups. BMI was not significantly associated with TOAST subtype. Three main functional outcomes measured by NIHSS, mRS, and BI scale also differed by etiologic stroke subtypes or overall mortality (Tables 2 and 3). NIHSS and mRS were higher in nonlacunar (CE or LAA) subtype. BI score was lowest in CE subtype. SAO patients had lowest overall mortality rate.

**Table 2:** Characteristics of overall population according to TOAST subgroup

	SAO	CE	LAA	*p*‐Value
	*n* = 1,077	*n* = 175	*n* = 1,364	
Gender, male, number (%)	660 (61.3)	78 (44.6)	833 (61.1)	<0.001
Age, mean (*SD*)	70.10 (12.51)	78.39 (10.19)	72.83 (12.61)	<0.001
BMI, mean (*SD*)	25.59 (17.31)	24.15 (3.53)	25.67 (19.02)	0.73
Systolic BP (Hg), mean (*SD*)	147.50 (18.53)	147.18 (22.56)	149.51 (19.11)	0.02
WBC (10^9^/L),mean (*SD*)	7.00 (2.19)	8.39(4.10)	7.84(2.74)	<0.001
Neutrophil (%), mean (*SD*)	65.53 (11.31)	74.75(12.53)	70.48(12.31)	<0.001
Lymphocyte (%), mean (*SD*)	27.17 (9.53)	20.49(10.62)	23.29(10.42)	<0.001
IL6 (pg/ml),mean (*SD*)	2.20 (1.26)	3.15(1.51)	2.69(1.41)	<0.001
CRP (mg/L), mean (*SD*)	1.69 (1.21)	2.47(1.37)	2.10(1.39)	<0.001
Cholesterol(mM), mean (*SD*)	4.56 (1.14)	4.28 (1.02)	4.66 (1.17)	<.001
HDL(mM), mean (*SD*)	1.07 (0.28)	1.04 (0.27)	1.05 (0.27)	.12
LDL(mM), mean (*SD*)	2.93 (0.85)	2.72 (0.76)	3.04 (0.88)	<.001
Smokers, number (%)	393 (36.5)	33 (18.9)	515 (37.8)	<.001
Drinking history (yes, %)	6 (3.4)	204 (15)	146 (13.6)	<.001
Hypertension history (yes, %)	841 (78.2)	116 (66.3)	1,088 (79.8)	<.001
Diabetes history (yes, %)	425 (39.5)	52 (29.7)	539 (39.5)	.037
AF history (yes, %)	36 (3.3)	163 (93.1)	68 (5.0)	<.001
CAD history (yes, %)	131 (12.2)	35 (20.0)	144 (10.6)	.001
NIHSS, mean (*SD*)	2.65 (4.92)	8.17 (8.00)	6.24 (6.14)	<.001
BI, mean (*SD*)	89.68 (15.33)	66.03 (31.35)	73.03 (25.85)	<.001
mRS, mean (*SD*)	1.52 (3.03)	2.69 (1.63)	2.51 (3.11)	<.001
Death, number (%)	3 (0.3)	18 (10.3)	71 (5.2)	<.0001

Note

Number shown were mean (*SD*) for continuous variables or number (%) for categorical variables.

Abbreviations: AF, Atrial Fibrillation; BI, Barthel Index; BMI, body mass index; BP, blood pressure; CAD, coronary artery disease; CE, cardioembolism; CRP, C‐reactive protein; HDL, high‐density lipoprotein; IL, interlink; LAA, large‐artery atherosclerosis; LDL, low‐density lipoprotein; mRS, modified Rankin Scale; NIHSS, National Institutes of Health Stroke Scale; SAO, small‐artery occlusion; *SD*, standard deviation; TOAST, the Trial of Org 10172 in Acute Stroke Treatment; WBC, white blood cell.

**Table 3:** Mortality in TOAST subtype and functional outcomes

	Total	Death	% of death	*p* Value
TOAST				
LAA + CE (nonlacunar)	1,539	89	6	.0001
SAO (lacunar)	1,077	3	0.3	
NIHSS				
Good	2,621	70	3	.0001
Severe (NIHSS ≥ 21)	50	23	32	
mRS				
Good (mRS ≤ 2)	1,931	22	1	.0001
Poor	609	70	10	
BI				
Good (BI ≥ 95)	851	5	1	.0001
Poor	1,689	87	5	

Abbreviations: BI, Barthel Index; CE, cardioembolism; LAA, large‐artery atherosclerosis; mRS, modified Rankin Scale; NIHSS, National Institutes of Health Stroke Scale; SAO, small‐artery occlusion; TOAST, the Trial of Org 10172 in Acute Stroke Treatment.

*p* Value was calculated using Fisher exact test.

### 3.2 Association between clinical characteristics and outcomes

**Figure 1:**
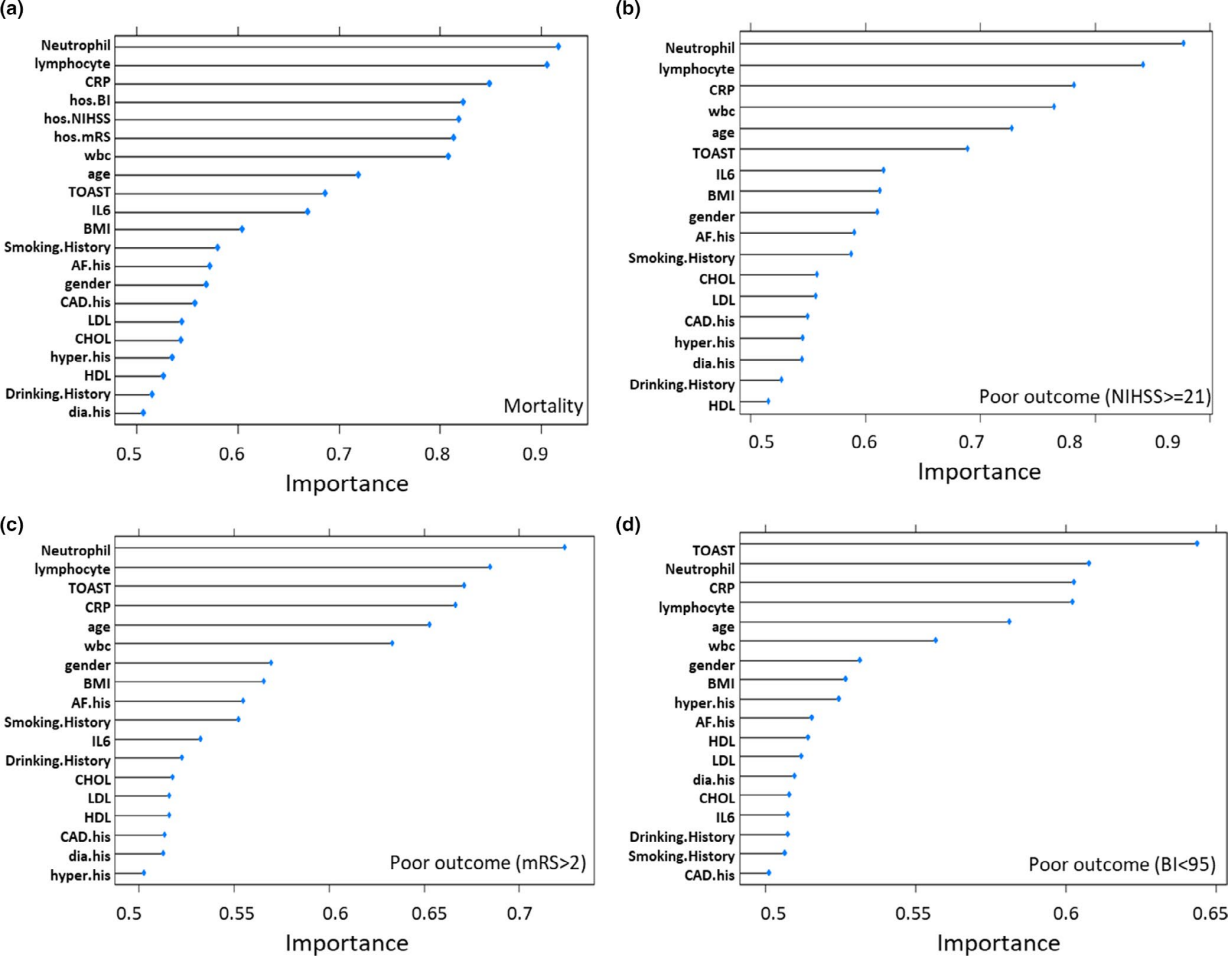
Importance score measured by area under receiver operating characteristics curve analyses using a Naïve Bayes regression model for each risk factor in the prediction of overall mortality (a), poor functional outcomes determined by NIHSS (b), mRS (c), and BI (d) in the overall stroke cohort

We evaluated traditional risk factors and inflammatory risk markers association with four types of outcomes including mortality (death) and three main functional outcomes defined by NIHSS, mRS, and BI score (Table 3). First, a Naive Bayesian classifier was used to estimate the risk associated with four types of outcomes for each risk factor presented by AUC. As shown in Figure 1a, number of neutrophil, lymphocyte, and level of CRP not only had higher prediction ability than conventional risk factors, but they also performed better than three score systems, as well as TOAST subtypes (nonlacunar versus lacunar) for mortality prediction. These three inflammatory risk markers performed best for the prediction of stroke severity defined by NIHSS and performed better than TOAST subtypes (Figure 1b). As for the disability prediction defined by mRS, counts of neutrophil and lymphocyte had best prediction power (Figure 1c). In contrast, for prediction of poor outcome defined by BI, TOAST classification had best performance. Neutrophil counts, CRP level, and lymphocyte counts only had modest prediction power; however, they were still better than convectional risk factors (Figure 1d. Overall, CRP level was a better predictor than IL‐6. Together, these data supported the role of inflammatory risk markers as strong predictors in the prediction of mortality as well as functional outcomes. In these models, age was the best predictor for poor outcomes among all conventional risk factors tested.

Furthermore, we applied stepwise feature selection and multivariable logistic models to evaluate the independence of these risk factors after correcting common conventional risk factors regarding association with four types of outcomes. As seen from Tables S1–S4, the stepwise regression model analysis led to various results of selected significant risk markers that were associated with each outcome. For instance, TOAST subtype and CRP level were consistently selected for all models with *p* value <.05. IL‐6 level was selected for all three models but not for BI‐defined outcome. In contrast, history of AF and history of CAD were not significantly selected for all type of outcomes. Total 13 risk factors including all five inflammatory risk markers and seven conventional risk markers (gender, age, systolic BP, smoking history, hypertension history, diabetes history and HDL or LDL) were selected with significant *p* value at least once in one of four models, thereby chosen to be tested in the final multivariable regression model for the independence. Because lymphocyte or neutrophil counts were not always selected in all four models, the neutrophil–lymphocyte ratio was used for final models as shown in Table 4. Table 4 shows the stroke unit characteristics independently associated with each outcome after mutual adjustment for other influential hospital characteristics. Among conventional risk factors, only age was consistently independently associated with four types of outcomes. Gender was independently associated with functional outcomes but not mortality in our study. The rest of conventional risk factors were only independently associated with one or two types of outcomes. For instance, smoking history or systolic blood pressure was only associated with BI or mRS‐defined outcome. Hypertension history was associated with NIHSS‐ and BI‐defined outcomes but diabetes history had zero independency. In contrast, CRP level and neutrophil–lymphocyte ratio were the best independent predictors among these tested inflammatory risk markers for all types of outcomes. IL‐6 and WBC count were independently associated with three outcomes but not BI‐defined outcome. In addition, TOAST subtype was another independent predictor for all four types of outcomes. Together, these results demonstrated that inflammatory risk markers had overall superior value to conventional risk factors.

**Table 4:** Outcome association analysis using multivariable logistic regression models

	Mortality				Poor outcome (NIHSS ≥ 22)				Poor outcome (mRS > 2)				Poor outcome (BI < 95)			
	OR	95% CI		*p*	OR	95% CI		*p*	OR	95% CI		P	OR	95% CI		P
Gender, male	0.85	0.47	1.54	.6	0.54	0.32	0.87	.01	0.66	0.51	0.84	.001	0.69	0.55	0.86	.001
Age	1.04	1.01	1.07	.01	1.04	1.02	1.06	.001	1.02	1.01	1.03	<.0001	1.01	1	1.02	.003
Systolic BP	1	0.99	1.01	.85	1	0.99	1.01	.81	1	1	1.01	.02	1	1	1.01	.13
WBC	1.19	1.11	1.27	<.0001	1.19	1.12	1.27	<.0001	1.08	1.03	1.14	.001	1.01	0.97	1.06	.63
HDL	0.4	0.14	1.08	.08	0.41	0.18	0.91	.03	0.65	0.44	0.95	.03	0.75	0.53	1.04	.09
Smoker	0.87	0.42	1.74	.69	0.92	0.51	1.64	.77	0.95	0.73	1.23	.67	1.32	1.06	1.65	.01
Hypertension history	0.62	0.35	1.11	.1	0.61	0.39	0.97	.03	0.91	0.71	1.16	.43	1.33	1.08	1.64	.01
Diabetes history	1.18	0.69	2.01	.54	0.74	0.47	1.14	.18	0.94	0.76	1.16	.56	0.89	0.74	1.06	.19
TOAST.SAO vs. nonlacunar	0.09	0.02	0.27	.0002	0.11	0.04	0.24	<.00001	0.22	0.17	0.28	<.0001	0.35	0.29	0.42	<.0001
CRP	1.5	1.24	1.82	<.0001	1.33	1.14	1.55	.0004	1.23	1.12	1.34	<.0001	1.13	1.04	1.23	.01
IL‐6	1.32	1.11	1.58	.002	1.24	1.06	1.45	.01	1.34	1.19	1.51	<.0001	1.07	0.95	1.21	.25
N.L.Ratio	1.13	1.08	1.2	<.00001	1.12	1.07	1.17	<.0001	1.07	1.03	1.1	<.0001	1.06	1.02	1.1	.001

Abbreviations: AF, atrial fibrillation; BI, Barthel Index; BMI, body mass index; BP, blood pressure; CAD, coronary artery disease; CI, confidence interval; CRP, C‐reactive protein; HDL, high‐density lipoprotein; IL, interlink; LDL, low‐density lipoprotein; mRS, modified Rankin Scale; N.L.Ratio, neutrophil–lymphocyte ratio; NIHSS, National Institutes of Health Stroke Scale; OR, odds ratio; SAO, small‐artery occlusion; TOAST, the Trial of Org 10172 in Acute Stroke Treatment; WBC, white blood cell.

^a^ Baseline score was measured when patients arrived at the hospital.

## 4 DISCUSSION

**Figure 2:**
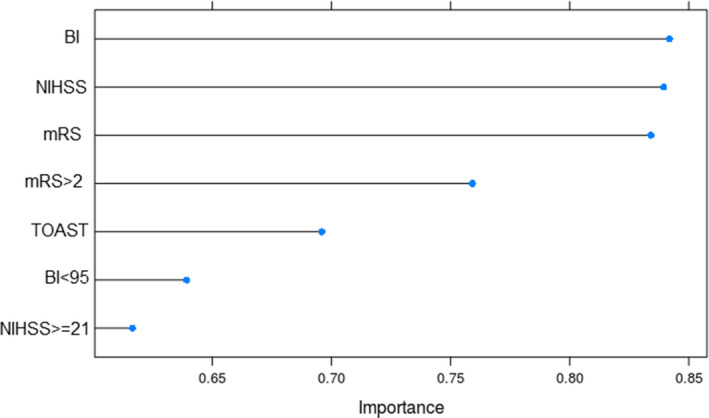
Importance score measured by area under receiver operating characteristics curve analyses using a Naïve Bayes regression model for three scale systems as continuous variables vs. categorical variables (poor outcomes were defined by mRS > 2, BI < 95, and NIHSS ≥ 21, respectively) as well as TOAST subtype in the overall stroke cohort

The inflammatory response plays an important role in the progression of stroke, and its modulation seems a promising strategy for neuroprotection and stroke prevention (Kelly et al., 2018). Inflammatory cells in vascular locations respond to known long‐term risk factors for human stroke such as hypertension, hyperlipidemia, diabetes mellitus, obesity, and smoking (Boehme et al., 2017). While various studies have demonstrated that inflammatory molecules could work as biomarkers for stroke diagnosis or prognosis, whether inflammatory parameters could be considered to modify stroke risk independent from conventional risk factors is a controversial topic (Bustamante et al., 2016; Simats et al., 2016; Whiteley et al., 2009). To our best knowledge, this study is the largest single retrospective study on Chinese population in which we interrogated the role for five inflammatory risk factors in the prediction of four most common stroke outcomes categories defined by overall mortality, NIHSS, mRS, and BI, taking into account the influence of common conventional risk factors. Our study revealed that the counts of neutrophil, lymphocyte, and CRP level were the best predictor for overall mortality, superior to three scaling systems. These three inflammatory molecules also had better prediction performance for outcome defined by NIHSS than TOAST. In the prediction of mRS‐defined outcome, the counts of neutrophil and lymphocyte remained to be the best performer and CRP level was inferior to TOAST only by a negligible margin. In contrast, TOAST subtype was the best predictor for BI‐defined outcome, whereas three inflammatory factors only had similar but modest impact. Interestingly, three scoring systems, as continues variables, had almost same impact in the prediction of mortality; nonetheless, when used as two level categorical variables, the prediction power was significantly different among three scales (Figure 2), further arguing the notion that when considering functional assessment to stroke outcome, no single outcome measure can describe or predict all dimensions of recovery and disability after acute stroke (Harrison et al., 2013; Kasner, 2006).

Among various inflammatory blood biomarkers, CRP and IL‐6 were two most studied in the literatures (Matsuo et al., 2016; VanGilder et al., 2014; Whiteley et al., 2009), some of which report conflicting results and this may reflect the complex physiology of CRP or IL‐6 and true differences between stroke subtypes and populations (Bustamante et al., 2014; Yu et al., 2017). In our study, the performance of CRP level for the prediction of all four types of outcomes was higher than IL‐6. In multivariate logistic model, after correcting conventional risk factors and TOAST subtype, both CRP and IL‐6 level were independent predictors for mortality, stroke severity, and mRS‐defined outcome. IL‐6 was not an independent predictor for BI‐defined outcome. In contrast, both neutrophil and lymphocyte counts only remained significance as independent predictor for mRS‐defined outcome. Neutrophil counts but not lymphocyte count was independent predictor for NIHSS‐defined outcome. Interestingly enough, when neutrophil–lymphocyte ratio was tested in the same models, the neutrophil–lymphocyte ratio turned to be an independent predictor for all types of outcome (Table 4), strongly supporting that neutrophil–lymphocyte ratio is a better predictor than neutrophil or lymphocyte alone (Brooks et al., 2014; Yu et al., 2018), even though the predictive power of neutrophil–lymphocyte ratio had no difference when compared with neutrophil counts alone.

Among conventional risk factors, age was the only one independently associated with four types of outcome in our study, consistent with many previous findings. Gender was associated with all three functional outcomes. Lipid level (HDL, LDL, and blood cholesterol) was highly correlated with one another, and HDL was significantly associated with two types of functional outcomes but not overall mortality in our study. Several studies showed that LDL and/or HDL level are independently associated with short‐term mortality (Reina et al., 2015; Zeljkovic et al., 2010). However, many of such studies did not take into account the impact from stroke subtype and inflammatory risk factors. One study even suggests high cholesterol levels are associated with improved long‐term survival after acute ischemic stroke (Markaki et al., 2014). The question whether lipid level is an independent predictor of stroke outcomes merits a more comprehensive study using a large cohort. In terms of disease history, history of hypertension was significantly associated with NIHSS‐ or BI‐defined outcomes in multivariable regression models, whereas history of diabetes was not independently associated with any outcomes.

One big limitation of our study was various extent of missing measurements of clinical risk factors. This resulted in a reduced patient number in our multivariable analysis models and may have resulted in bias, even though we replaced missing value for certain continuous variable such as CRP and IL‐6. We did not apply any statistical models for replacing those missing categorical variables. For instance, overall mortality in our study was only 3% (96/3013), which may lead to an issue of unbalanced sample size in regression models. Moreover, this was a retrospective study, and our findings warrant a validation in a future prospective study.

## 5 CONCLUSIONS

In this large retrospective cohort of stroke patients, we found blood markers of the acute inflammatory response were associated with mortality and poor outcome defined by three different scaling systems. Neutrophil–lymphocyte ratio and CRP level were the best independent predictors among five markers tested in this study, after adjustment for confounding factors, including conventional risk factors and TOAST subtype. TOAST subtype was not associated with mRS‐defined outcome. Different stroke outcome scaling system should be used with caution.

## CONFLICT OF INTEREST

All the authors declare that there is no conflict of interest.

## AUTHOR CONTRIBUTION

Ling‐Yun Liu and Yun‐Hua Yue contributed to the conception and design of the study. Wen‐Hao Yang, Xu‐Shen Xu, Hong‐Xian Sun, and Liang Hu contributed to the acquisition of data. Xiao‐Guang Zhang and Jie Xue contributed to the analysis of data. Xiao‐Guang Zhang wrote the manuscript. All authors contributed to manuscript revision, read, and approved the submitted version.

### Peer Review

The peer review history for this article is available at https://publons.com/publon/10.1002/brb3.1922.

## Supporting information

Table S1‐S4

## Data Availability

The datasets used and/or analyzed during the current study are available from the corresponding author on reasonable request.
