# Supplementary material for: Inflammatory markers as independent predictors for stroke outcomes
Source: Brain Behav. 2020 Dec 12;11(1):e01922. doi: 10.1002/brb3.1922 (PMC7821567; doi:10.1002/brb3.1922)
Supplement: Supplementary file 1 — Table S1‐S4 [file BRB3-11-e01922-s001.docx]

**Supplemental Table 1 Stepwise feature selection for the mortality regression model**

|  | OR | 95% C.I. | P value |
| --- | --- | --- | --- |
| Age | 1.00 | 1.0001-1.0012 | 0.03 |
| WBC | 1.02 | 1.0136-1.0193 | <0.0001 |
| Lymphocyte | 1.00 | 0.998-0.9995 | 0.002 |
| Smoker | 0.98 | 0.9679-0.9987 | 0.03 |
| Drinking History | 1.02 | 0.9951-1.0381 | 0.13 |
| Hypertension History | 0.98 | 0.9623-0.9926 | 0.004 |
| CAD History | 1.02 | 0.997-1.0376 | 0.10 |
| TOAST.SAO vs non-lacunar | 0.98 | 0.9664-0.9926 | 0.002 |
| CRP | 1.01 | 1.0073-1.0193 | <0.0001 |
| IL-6 | 1.03 | 1.0238-1.0395 | <0.0001 |

WBC, white blood cell; IL, interlink; CRP, C-reactive protein; CAD, coronary artery disease; TOAST, the Trial of Org 10172 in Acute Stroke Treatment; SAO, small-artery occlusion; OR, odds ratio. C.I., confidence interval.

**Supplemental Table 2 Stepwise feature selection for the regression model of NIHSS defined outcome**

|  | OR | 95% C.I. | P value |
| --- | --- | --- | --- |
| Gender.Male | 0.97 | 0.9509-0.9841 | 0.0002 |
| Age | 1.00 | 1.0002-1.0016 | 0.01 |
| WBC | 1.02 | 1.0149-1.0221 | <0.0001 |
| Neutrophil | 1.00 | 1.001-1.0027 | <0.0001 |
| LDL | 0.99 | 0.9793-0.9979 | 0.02 |
| Hypertension History | 0.97 | 0.9525-0.9897 | 0.003 |
| Diabetes History | 0.98 | 0.9638-0.9958 | 0.01 |
| AF History | 1.02 | 0.9933-1.0488 | 0.14 |
| CAD History | 1.02 | 0.9968-1.047 | 0.09 |
| TOAST.SAO vs non-lacunar | 0.96 | 0.9455-0.9774 | <0.0001 |
| CRP | 1.01 | 1.007-1.0217 | 0.0001 |
| IL-6 | 1.03 | 1.0211-1.0406 | <0.0001 |

WBC, white blood cell; IL, interlink; CRP, C-reactive protein; LDL, low-density lipoprotein; AF, Atrial Fibrillation; CAD, coronary artery disease; NIHSS, National Institutes of Health Stroke Scale. TOAST, the Trial of Org 10172 in Acute Stroke Treatment; SAO, small-artery occlusion; OR, odds ratio. C.I., confidence interval.

**Supplemental Table 3 Stepwise feature selection for the regression model of mRS defined outcome**

|  | OR | 95% C.I. | P value |
| --- | --- | --- | --- |
| Gender.Male | 0.93 | 0.8962-0.9578 | <0.0001 |
| Age | 1.00 | 1.0018-1.0045 | <0.0001 |
| Systolic BP | 1.00 | 1.0001-1.0012 | 0.03 |
| WBC | 1.01 | 1.0026-1.0163 | 0.01 |
| Neutrophil | 1.01 | 1.004-1.0072 | <0.0001 |
| HDL | 0.94 | 0.8881-0.9954 | 0.03 |
| AF History | 1.05 | 0.9959-1.1036 | 0.07 |
| TOAST.SAO vs non-lacunar | 0.81 | 0.7882-0.8396 | <0.0001 |
| CRP | 1.03 | 1.0178-1.0464 | <0.0001 |
| IL-6 | 1.05 | 1.0331-1.071 | <0.0001 |

WBC, white blood cell; IL, interlink; CRP, C-reactive protein; HDL, high-density lipoprotein; AF, Atrial Fibrillation; mRS, modified Rankin Scale. TOAST, the Trial of Org 10172 in Acute Stroke Treatment; SAO, small-artery occlusion; OR, odds ratio. C.I., confidence interval.

**Supplemental Table 4 Stepwise feature selection for the regression model of BI defined outcome**

|  | OR | 95% C.I. | P value |
| --- | --- | --- | --- |
| Gender.Male | 0.93 | 0.8901-0.9709 | 0.001 |
| Age | 1.00 | 1.0012-1.0043 | 0.001 |
| BMI | 1.00 | 0.9979-1.0001 | 0.08 |
| Lymphocyte | 1.00 | 0.9939-0.9978 | <0.0001 |
| CHOL | 1.01 | 0.9969-1.031 | 0.11 |
| HDL | 0.93 | 0.8653-0.9993 | 0.05 |
| Smoker | 1.05 | 1.0091-1.1003 | 0.02 |
| Hypertension History | 1.05 | 1.0107-1.0979 | 0.01 |
| AF History | 0.95 | 0.891-1.0023 | 0.06 |
| TOAST.SAO vs non-lacunar | 0.79 | 0.7656-0.8227 | <0.0001 |
| CRP | 1.02 | 1.0095-1.0402 | 0.001 |

BMI, body mass index; BP, blood pressure; CRP, C-reactive protein; HDL, high-density lipoprotein; AF, Atrial Fibrillation; CAD, coronary artery disease; BI, Barthel Index; TOAST, the Trial of Org 10172 in Acute Stroke Treatment; SAO, small-artery occlusion; OR, odds ratio. C.I., confidence interval.
